# Supplementary material for: Aerodynamic Ground Effect in Fruitfly Sized Insect Takeoff
Source: PLoS One. 2016 Mar 28;11(3):e0152072. doi: 10.1371/journal.pone.0152072 (PMC4809487; doi:10.1371/journal.pone.0152072)
Supplement: S1 Appendix — See file S1_Appendix.pdf. (PDF) [file pone.0152072.s001.pdf]

S1 Appendix. Numerical validation of the ground plane modelling using the volume penalization method.

A numerical study of the ground effect during hovering flight was carried out by Gao and Lu [1] in the two-dimensional approximation. In this section, we compare with some of their results.

All quantities in [1] are presented in a non-dimensional form, and we follow the same conventions. The wing cross-section is an ellipse, as schematically shown in Fig. 1. Its major axis (chord length) is $c = 1$, and its minor axis is equal to 0.25. The motion of the ellipse centre in the horizontal direction (x direction) is given by

$$x_c(t) = A_m \cos(2\pi t/T), \tag{1}$$

where $A_m = 1.25$, and we fix the dimensionless stroke time period to $T = 2\pi A_m$. The vertical coordinate of the ellipse centre is constant in time. The angle between the major axis and the horizontal axis varies according to

$$\alpha(t) = \alpha_0 - \alpha_m \sin(2\pi t/T), \tag{2}$$

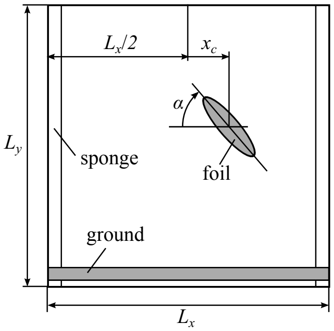

Figure 1. Schematic drawing of the setup.

where $\alpha_0 = 90^\circ$ and $\alpha_m = 45^\circ$.

The ground surface is horizontal (x direction), so that the distance D between the ellipse centre and the ground remains constant in time. In the present study, we vary this parameter between 1 and 6.

The dimensionless density of the fluid is $\rho = 1$. The dimensionless kinematic viscosity is equal to $\nu = 10^{-2}$ yielding the Reynolds number

$$Re = \frac{Uc}{\nu} = 100, \qquad \text{where} \quad U = \frac{2\pi A_m}{T} = 1. \tag{3}$$

Our method solves the three-dimensional incompressible Navier–Stokes equations. The two-dimensional flow is therefore modelled by imposing the initial and boundary conditions constant in the direction perpendicular to the flow plane (z direction). The domain size in this direction is $L_z = 1$.

In the xy plane, the computational domain size is equal to $L_x \times L_y = 12 \times 12$. The number of grid points is equal to $N_x \times N_y = 512 \times 512$ (*low resolution*) or 1024×1024 (*high resolution*). In the low resolution simulations, the grid step size $\Delta x \approx 0.0234$ is comparable to the lowest value reported in [1] ($\Delta x = 0.025$).

The ground is modelled as a solid layer of width 0.2. Its top surface is at distance D below the centre of the ellipse. Smoothing of the penalization mask function (erf, $3\Delta x$ inwards and $3\Delta x$ outwards, see [2]) is only applied to the ellipse, not to the ground.

The penalization parameter, both for the ellipse and for the ground, is equal to $\varepsilon = 10^{-3}$ in the low-resolution simulations and $2.5 \cdot 10^{-4}$ in the high-resolution simulations. A 'vorticity sponge' forcing term [2] is introduced in the momentum equation equation in order to weaken the effect of the periodic boundary conditions in x. It is applied in two vertical layers of thickness equal to $32\Delta x$, and its penalization parameter is equal to $\varepsilon_{sponge} = 0.1$.

The nearest distance to the ground in [1] is $D = 1$. Fig. 2(a) displays the time evolution of the vertical force coefficient C_V, obtained by normalizing the vertical force F_V,

$$C_V = \frac{F_V}{0.5\rho U^2 c L_z}. \tag{4}$$

where $U = 2\pi A_m/T$. In the figures, the time t is normalized to the stroke period T.

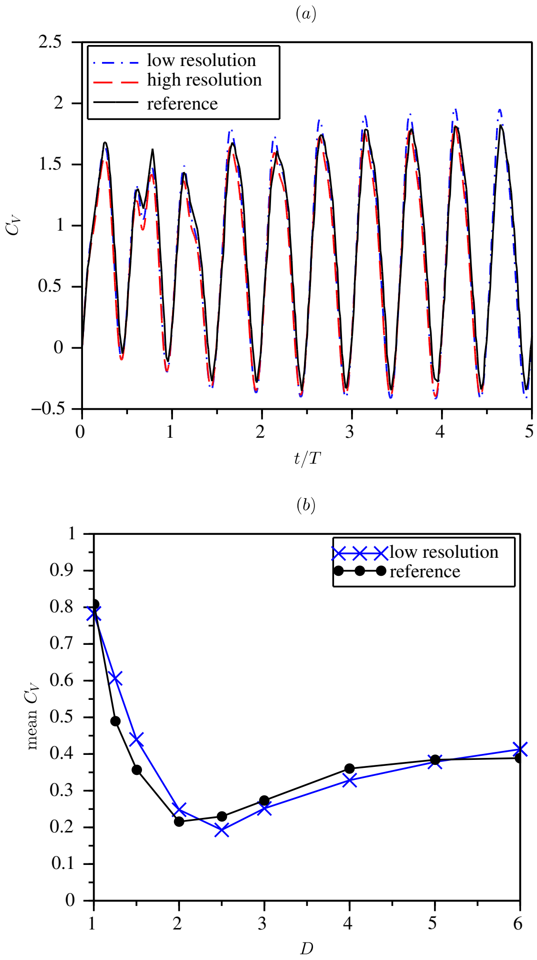

Figure 2. Results of the numerical simulations. (a) Time evolution of the vertical force coefficient C_V at the distance from the ground $D = 1$. (b) Mean vertical force coefficient versus distance from the ground.

After $t/T = 1$, oscillations of C_V are mainly described by the second harmonic. They become apparently periodic after $t/T = 4$.

A series of simulations has been carried out in order to determine how the time averaged force coefficients depend on D. Their parameters correspond to the low resolution, as defined above. As shown in Fig. 2(b), the minimum of C_V is observed in our simulations at about the same D as in [1]. We conclude that, in this two-dimensional validation case, our results are in reasonable agreement with the reference [1].

References

1. Gao T, Lu X. Insect normal hovering flight in ground effect. Physics of Fluids. 2008;20:087101.

2. Engels T, Kolomenskiy D, Schneider K, Sesterhenn J. Numerical simulation of fluid-structure interaction with the volume penalization method. Journal of Computational Physics. 2015;281:96–115.
