# Supplementary material for: Aerodynamic Ground Effect in Fruitfly Sized Insect Takeoff
Source: PLoS One. 2016 Mar 28;11(3):e0152072. doi: 10.1371/journal.pone.0152072 (PMC4809487; doi:10.1371/journal.pone.0152072)
Supplement: S2 Appendix — See file S2_Appendix.pdf. (PDF) [file pone.0152072.s002.pdf]

S2 Appendix. Influence of the domain size in the vertical direction.

In our numerical simulations of takeoffs, the insect approaches the top of the computational domain as time increases. The simulations have to be stopped before the finite domain size begins to influence the results significantly. To quantify this effect, we compare the aerodynamic forces and power in the voluntary takeoff, computed using two different vertical domain sizes, $L_z = 6R$ and $L_z = 8R$. We consider the OGE case in which the bee is nearer to the top of the domain.

Figure 1 shows the relative error for the aerodynamic force magnitude, $(F_{abs,ave,OGE,6R} - F_{abs,ave,OGE,8R})/F_{abs,ave,OGE,8R} \cdot 100\%$, and for the aerodynamic power, $(P_{ave,OGE,6R} - P_{ave,OGE,8R})/P_{ave,OGE,6R} \cdot 100\%$. The horizontal axis shows the distance from the insect body point of reference to the 'ceiling', *i.e.*, the penalization layer at the top of the domain, normalized to the wing length R. We use the data for the case $L_z = 8R$ to calculate this distance. When the domain size is equal to $L_z = 6R$, the duration of the takeoff spans four complete wingbeats, and the wings leave the domain during the 5th wingbeat. Hence, the 4th wingbeat is the most sensitive to the aerodynamic interaction with domain boundary. The maximum difference for the force and for the power is 0.6% and 1.6%, respectively. During the first three wingbeats, the error is less than 0.5%.

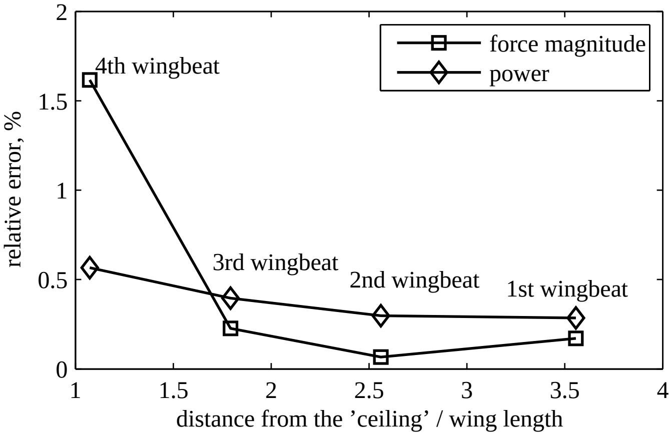

Figure 1. Influence of the domain size in the vertical direction. Relative error is calculated for the aerodynamic force magnitude and the aerodynamic power, and plotted versus the distance from the 'ceiling' of the flow domain.
